# Real-Time Imaging of NADPH Oxidase Activity in Living Cells Using a Novel Fluorescent Protein Reporter

**DOI:** 10.1371/journal.pone.0063989

**Published:** 2013-05-21

**Authors:** Rituraj Pal, Poulami Basu Thakur, Shumin Li, Charles Minard, George G. Rodney

**Affiliations:** 1 Department of Molecular Physiology and Biophysics, Baylor College of Medicine, Houston, Texas, United States of America; 2 Dan L. Duncan Institute for Clinical and Translational Research, Baylor College of Medicine, Houston, Texas, United States of America; University of Illinois at Chicago, United States of America

## Abstract

Production of reactive oxygen species (ROS) has been implicated in the pathology of many conditions, including cardiovascular, inflammatory and degenerative diseases, aging, muscular dystrophy, and muscle fatigue. NADPH oxidases (Nox) have recently gained attention as an important source of ROS involved in redox signaling. However, our knowledge of the source of ROS has been limited by the relatively impoverished array of tools available to study them and the limitations of all imaging probes to provide meaningful spatial resolution. By linking redox-sensitive GFP (roGFP) to the Nox organizer protein, p47^phox^, we have developed a redox sensitive protein to specifically assess Nox activity (p47-roGFP). Stimulation of murine macrophages with endotoxin resulted in rapid, reversible oxidation of p47-roGFP. In murine skeletal muscle, both passive stretch and repetitive electrical stimulation resulted in oxidation of p47-roGFP. The oxidation of p47-roGFP in both macrophages and skeletal muscle was blocked by a Nox specific peptide inhibitor. Furthermore, expression of p47-roGFP in p47^phox^ deficient cells restored Nox activity. As Nox has been linked to pathological redox signaling, our newly developed Nox biosensor will allow for the direct assessment of Nox activity and the development of therapeutic Nox inhibitors.

## Introduction

Free radicals and other reactive oxygen species (ROS) are produced in a wide range of physiological processes and have long been associated with inflicting biological damage. Generation of superoxide and other downstream ROS by NADPH oxidase (Nox) has long been ascribed to phagocytes. More recently, several homologs of the phagocyte Nox (gp91^phox^/Nox2) have been found (Nox1, Nox3, Nox4, Nox5, DuoX1, and DuoX2) in other tissues, including endothelial cells, vascular smooth muscle cells, macrophages, adventitial fibroblasts, cardiac myocytes, fibroblasts, adipocytes, stem cells, as well as skeletal muscle [1]. Increased production of ROS from the non-phagocytic Noxs has been implicated in ischemia reperfusion, hypertension, heart failure, atrial fibrillation, Alzheimer's and Parkinson's disease, muscular dystrophy and muscle fatigue.

Customary redox measurements of Nox activity use colorimetric or luminescent probes that require cell lysis and addition of NADPH, FAD, and recombinant cytosolic factors, making measurements prone to artifacts [2], [3] and impractical in living cells. Redox-sensitive fluorescent dyes such as DCFH have been used to detect oxidant generation within living cells. However, these dyes are prone to movement and bleaching artifacts, are non-reversible, lack specificity for the site of ROS generation, display low sensitivity, and even promote artificial ROS formation [4], [5]. Due to the potential participation of Nox in a variety of diseases, there is a need to selectively measure ROS production from the Nox enzyme complex.

The most promising tools for dynamic and site-specific assessment of redox potential are genetically encoded redox probes based on green fluorescent protein [6]. These genetically encoded biosensors have been targeted to specific sub-cellular compartments, including the mitochondria, endoplasmic reticulum, and plasma membrane. They have also been fused with peroxidases (Orp1-roGFP) or Glutaredoxin 1 (Grx1-roGFP) to improve the specificity of the probe. Another critical advantage of these probes is that they are ratiometric by excitation, minimizing measurement errors due to variable concentration, photobleaching, or movement artifacts. Using redox-sensitive GFP (roGFP) targeted to the mitochondria (mito-roGFP) we have previously shown that increased contractile activity promotes ROS formation not from the mitochondria but potentially via Nox2 in skeletal muscle [7].

The activation of the Nox2 complex requires the association of cytosolic components (p47^phox^, p67^phox^, and Rac1) with the membrane bound p22^phox^ and Nox2 [8]. Phosphorylation of p47^phox^ facilitates the recruitment and binding of Rac1 and p67^phox^ to the Nox2 complex, resulting in superoxide production. We have now developed a redox sensitive protein to locally assess Nox activity by fusion of roGFP with p47^phox^ (p47-roGFP). This novel Nox biosensor allows for dynamic measurement of Nox dependent ROS production with high spatial and temporal resolution under physiologically relevant processes in living cells.

## Materials and Methods

### Ethical Statement

Baylor College of Medicine's Institutional Animal Care and Use (IACUC) reviewed and approved all animal procedures performed in these studies (Protocol #AN-5829), which were aligned with the recommendations in the Guide for the Care and Use of Laboratory Animals of the National Institutes of Health.

### Cloning of the p47-roGFP biosensor construct

Human p47^phox^ was amplified by PCR using a previously described expression vector, pcDNA3.1-p47^phox^ [9] as a template. HindIII and SpeI restriction sites (underlined in primer sequence) were added to the forward (5′-CTCGAGCTC**AAGCTT**ATGGGGGACACCTTCATCCGT-3′) and reverse (5′-CACCACCTGAACCACC **ACTAGT**GACGGCAGACGCCAGCTTCCG-3′) primers, respectively. The start of a 30-amino-acid linker sequence (Gly-Gly-Ser-Gly-Gly)_6_ preceding roGFP2 was added to the 5′- end of the reverse primer (indicated by the broken underline).

The coding sequence of roGFP2 with the linker fused to its N-terminus was PCR amplified from the pLPCX-Grx1-roGFP2 plasmid construct [10], using the primers 5′- CGGAAGCTGGCGTCTGCCGTC **ACTAGT**GGTGGTTCAGGTGGTG-3′ and 5′- TCTAGAGTC**GCGGCCGC**TTTACTTGTACAGCTCGTCCAT-3′ with the SpeI and NotI restriction sites on the forward and reverse primers (as underlined), respectively. The end of the p47^phox^ sequence was added to the 3′ end of the forward primer, as indicated by the broken underline, to facilitate ligation with the amplified p47^phox^ sequence. The PCR products were purified, ligated and cloned into the pcDNA3.1 vector (Invitrogen) using restriction sites HindIII and NotI, to generate the recombinant pcDNA3.1-p47-roGFP expression vector.

### Cell culture of living RAW264.7 cells and primary macrophages

RAW264.7 cells (ATCC) were grown in DMEM (Gibco) supplemented with 10% heat inactivated fetal bovine serum (FBS, Atlanta Biologicals), 2 mM L-glutamine, 100 U/ml penicillin and 100 mg/ml streptomycin (Gibco).Cells were seeded ∼10000 cells/well on 96-well plates (Costar) and transiently transfected with p47-roGFP using X-tremeGENE HP DNA Transfection Reagent (Roche) at a ratio of 3∶1 (Reagent to DNA).

Wild-type (C57Bl/6J), Nox2^−/y^ (B6.129S-Cybbtm1Din/J), and p47phox^−/−^ (B6(Cg)-Ncf1m1J/J) mice 6–8 weeks of age from The Jackson Laboratory (Bar Harbor, MA) were euthanized by an overdose of anesthetic (isoflurane) followed by rapid cervical dislocation. Macrophages were isolated from spleens as previously described [11]. Briefly, spleens were collected for preparation of a single-cell suspension using 70 µm cell strainers and enrichment of mononuclear cells on a density gradient (Histopaque-1077; Sigma). Macrophages were further enriched through their adhesion to plastic culture dishes and maintained in RPMI supplemented with 100 U/ml penicillin and 100 mg/ml streptomycin (Gibco), 4 mM L-Glutamine, 1 mM Sodium pyruvate, 1% Non-essential amino acids, 1% RPMI vitamins, 50 µM β mercaptoethanol, and 10% heat inactivated FBS. Cells were transiently transfected with p47-roGFP using jetPEI-Macrophage DNA transfection reagent (Polyplus transfection).

### In-Vivo Electroporation

Transfection of redox sensitive GFPs into mouse flexor digitorum brevis (FDB) was performed as previously described [7]. Briefly, male wild-type, Nox2^−/y^, and p47^phox−/−^ mice, 8–10 weeks of age were anesthetized with isoflurane (2%) in accordance with National Institutes of Health guidelines and approved by the Institutional Animal Care and Use Committee of Baylor College of Medicine. Hyaluronidase (10 µl of 0.5 mg/ml) dissolved in sterile saline was injected subcutaneously into the right foot pad followed by 30–40 µg of rDNA in PBS 1.5 hrs later. Two electrodes were placed subcutaneously at the proximal and distal tendons to deliver 20 pulses of 150 V, 20 ms in duration at a frequency of 1 Hz with a square pulse stimulator (S48; Grass Technologies, West Warwick, RI). FDB muscle fibers were isolated 6–8 days later. Typically, the right foot was electroporated, while the left foot served as a contra-lateral control.

### Isolation of FDB fibers

Mice were deeply anesthetized by isofluorane (2%) inhalation and euthanized by rapid cervical dislocation. FDB muscles were surgically isolated and incubated in minimal essential media containing 0.1% gentamycin and 0.4% Collagenase A (Roche Applied Science, Indianapolis, IN) at 37°C for 1.5–2.0 h. To release single fibers, FDB muscles were then triturated gently in serum containing media (10%, Atlanta Biologicals) without collagenase and incubated in 5% CO2 at 37°C until used, typically 12–36 h later. On the day of experiments, fibers were plated on ECM gel from Engelbreth-Holm-Swarm murine sarcoma (Sigma, St. Louis, MO) coated 96 well culture dishes (Costar, Corning Incorporated Life Sciences, Lowell, MA).

### Loading of single FDB fibers with DCFH-DA

6-carboxy-2′,7′-dichlorodihydrofluorescein diacetate (DCFH-DA) (Invitrogen, Carlsbad, CA) was prepared in dimethyl sulfoxide (DMSO)/pluronic. Culture dishes containing isolated FDB fibers were washed with Ringer's solution containing DCFH-DA (5 µM) for 30 min at room temperature. The fibers were then washed with Ringer's solution and the dye allowed to de-esterify for 20 min prior to fluorescence microscopy. To prevent light induced oxidation of DCFH, all cell-loading was performed in the dark.

### Passive stretch of single FDB fibers

Single enzymatically dissociated FDB myofibers without tendons were attached to glass micro-holders coated with extracellular matrix proteins and set at a resting sarcomere length of 2.0 µm. Fibers were passively stretched (no electrical stimulation) to 120% (2.4 µm) of resting length.

### Electrical stimulation of single FDB fibers

Isolated FDB myofibers were perfused with Ringer's solution containing (in mM): 140 NaCl, 4.0 KCl, 1.0 MgSO4, 5.0 NaHCO3, 10.0 glucose, 10.0 HEPES, pH 7.3, for 2 min to obtain a baseline fluorescence of p47-roGFP or Grx1-roGFP2 (. Subsequently, intermittent trains (0.25 Hz, 500 ms train duration) of tetanic stimulation (80 Hz) were delivered to isolated FDBs for 15 min via a custom-built perfusion/electrical stimulation chamber (Four Hour Day Foundation, Towson, MD). A gravity fed perfusion system allowed rapid changing of perfusate during imaging.

### Microscopy

The redox sensitive biosensors were excited at 403/12 nm and 470/20 nm using a Sutter Lamda DG-5 Ultra high speed wavelength switcher, and the emission intensity was collected at 535/48 nm on a charge coupled device (CCD) Camera (Rolera-XR, QImaging, Tucson, AZ) attached to an Axio Observer (Zeiss) inverted microscope (40× H_2_O objective, 1.2 NA). At the end of stimulus, LPS or tetanic electrical stimulation, cells were exposed to 1 mM H_2_O_2_ for 5 min to obtain a maximal oxidation, followed by dithiothreitol (DTT, 10 mM) for 5 min to obtain maximal reduction of p47-roGFP. Regions of interest (ROI) were drawn on the cell and in the background. Ratio images (403/470) were created by subtracting the mean background ROI from the mean cell ROI.

Dichlorofluorescein (DCF) fluorescence was excited at 480 nm via a Sutter Lamda DG-5 Ultra high speed wavelength switcher, and emission intensity was collected at 510 nm at a rate of 0.1 Hz. The time dependent (no LPS or no electrical stimulation) photo-oxidation of DCF was monitored from each condition. At the end of the stimulus, either LPS or tetanic electrical stimulation, DCFH-loaded cells were exposed to 1 mM H_2_O_2_ to obtain a maximal rate of DCFH oxidation for each cell. Since the amount of DCFH loaded is variable from cell to cell, the maximal rate of DCF fluorescence in the presence of 1 mM H_2_O_2_ was used to normalize the rate of change of DCF fluorescence for each cell.

To localize endogenous p47^phox^ fibers were fixed with 4% paraformaldehyde and permeabilized with 0.1% triton as previously described [12]. Fibers were then incubated in 8% goat serum (Jackson Immuno-Rearch) for 1 h at 4°C followed by incubation overnight at 4°C with a rabbit polyclonal antibody against p47^phox^ (1∶100, Upstate) in 2% donkey serum followed by labeling with an Alexa488-conjugated donkey anti-rabbit secondary antibody (1∶100, Invitrogen). Fibers were then washed three times with PBS and blocked with 8% donkey serum followed by incubation with a monoclonal antibody against RyR1 (1∶100, Pierce) overnight. Fibers were then washed three times with PBS containing 2% donkey serum at room temperature, followed by incubation with Cy5-conjugated anti-mouse IgG (1∶100, Millipore) overnight at 4°C. To localize p47-roGFP single living fibers were co-stained with the membrane dye FM4-64® (Invitrogen) according to the manufacturer's protocol.

### Data Analysis

Data are reported as mean ± SEM, unless otherwise specified. Statistical analysis was performed in Origin Pro (OriginLab Corporation, Northhampton, MA). To evaluate the time dependence of DCF and p47-roGFP response a general linear mixed model assuming a first-order autoregressive matrix of correlated error terms was used to compare the log-transformed ratios observed over time with 0. The model included cubic effects for time, cell line type and all interactions. P-values were adjusted using the FDR method. Statistical significance was assessed at the 0.05 level.

## Results

### p47-roGFP facilitates imaging of redox changes during immune cell activation

**Figure 1:**
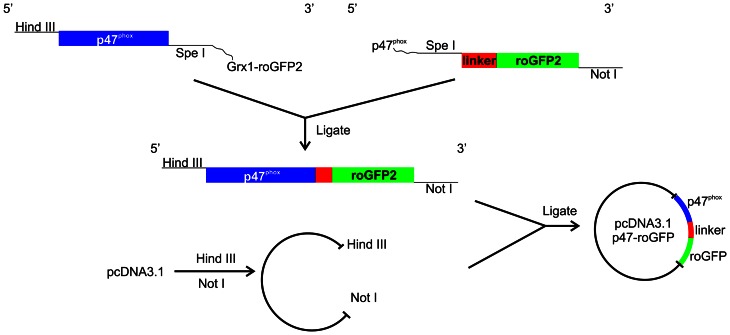
Construction of the Nox biosensor p47-roGFP. The coding sequences of human p47^phox^ and roGFP2, carrying a 30-amino-acid linker, were PCR amplified from pcDNA3.1-p47^phox^ and pLPCX-Grx1-roGFP2, respectively as described in Materials and Methods. The forward and reverse primers for the two reactions were designed to incorporate HindIII, SpeI and NotI restriction sites. Both PCR products were purified and ligated to form a linear p47^phox^-linker-roGFP expression cassette, which was cloned into the pcDNA3.1 vector using HindIII and NotI restriction sites.

**Figure 2:**
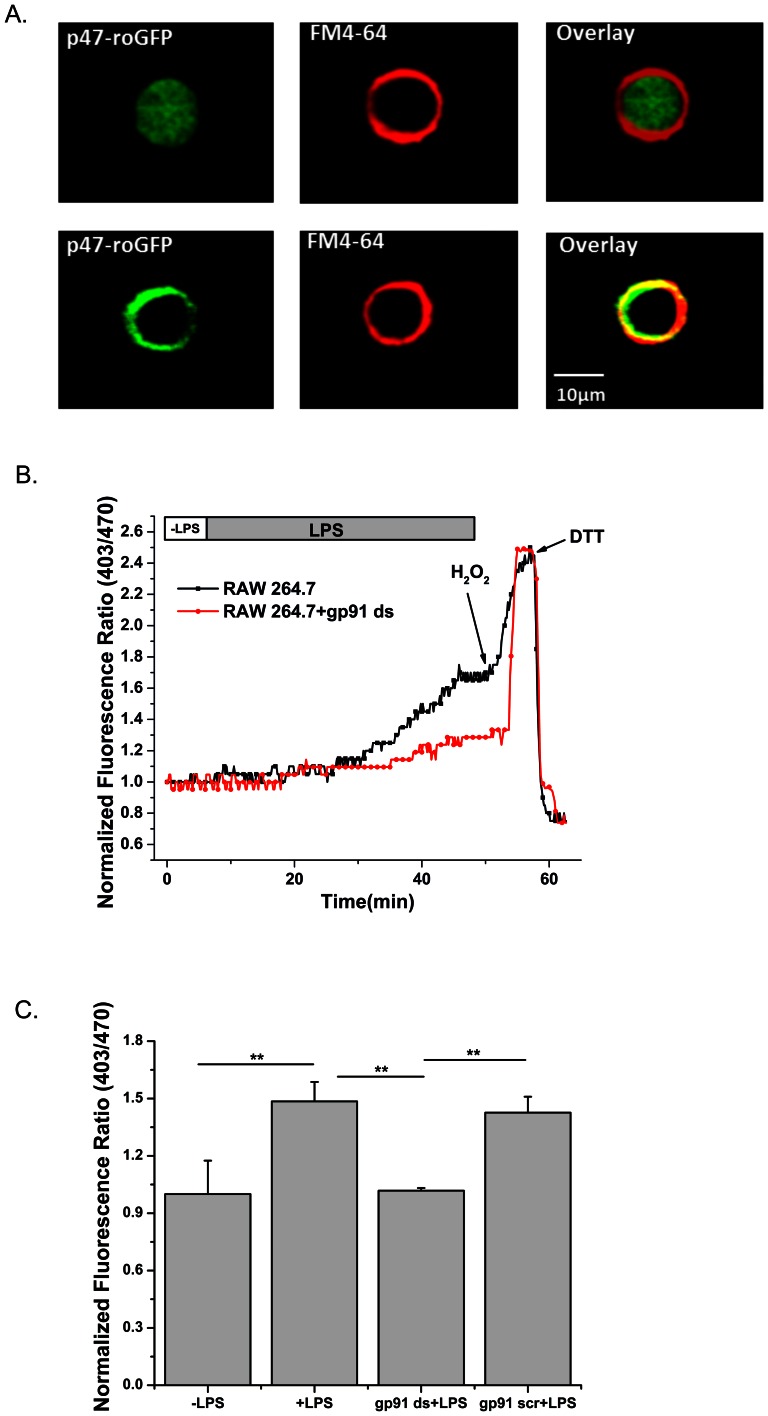
p47-roGFP allows for live-cell imaging of redox changes during RAW264.7 cell activation. (*A*) Representative confocal image of RAW264.7 cells transfected with p47-roGFP shows cytosolic distribution of p47-roGFP in control cells (−LPS). Upon LPS-mediated activation (20 ng/ml, 45 min) p47-roGFP localized with the membrane dye FM4-64®. (B) Representative time course of LPS-induced p47-roGFP oxidation. Biosensor oxidization occurred dynamically upon LPS-induced activation (black squares), while in the presence of the Nox peptide inhibitor gp91ds (5 µm, 60 min) minimal oxidation occurred (red circles). After 45 min of LPS, cells were treated with 1 mM H_2_O_2_ to maximally oxidize followed by addition of 10 mM DTT to maximally reduce the biosensors. (C) Average (±SEM) of 9 cells for each condition from (B) during the last 3 minutes of LPS. ** p<0.01 (Tukey statistical analysis for p value).

To measure sub-cellular ROS formation specifically from Nox we fused human p47^phox^ to the N terminus of roGFP2 via a 30-amino-acid linker (Gly-Gly-Ser-Gly-Gly)_6_ (Fig. 1). The production of superoxide from macrophages in response to endotoxin such as lipopolysaccharide (LPS) is an important innate immune response. LPS activates the regulatory subunit p47^phox^, resulting in translocation of p47^phox^ from the cytoplasm to the cell membrane and activation of Nox2 [13]. To investigate activation and redox-sensing ability of p47-roGFP in living cells, we transiently expressed p47-roGFP in RAW264.7 macrophages. Stimulation of RAW264.7 cells with LPS (20 ng/ml) resulted in translocation of p47-roGFP from the cytosol to the cell membrane (Fig. 2A) and a 1.5 fold increase in oxidation of the sensor, which plateaued after 45 minutes (Fig. 2B). The plateau formed upon LPS activation was not due to complete oxidation of the biosensor, as addition of H_2_O_2_ resulted in further oxidation of p47-roGFP (Fig. 2B). Furthermore, addition of DTT resulted in reduction of p47-roGFP (Fig. 2B). Use of the Nox peptide inhibitor gp91ds-TAT [14] (gp91ds) prevented the LPS induced oxidation of p47-roGFP (Fig. 2B–C) while a scrambled control peptide (gp91scr) had no effect (Fig. 2C), suggesting that our biosensor is specific for Nox activity.

**Figure 3:**
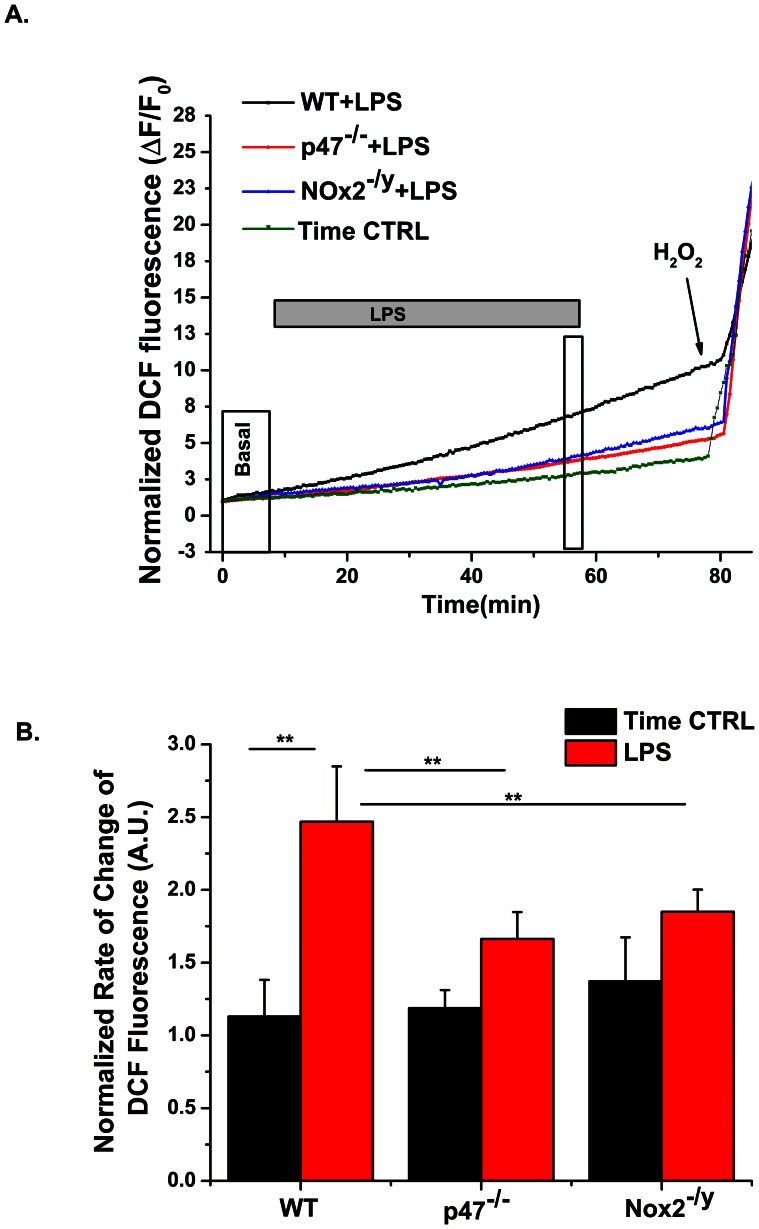
LPS-induced stimulation increases the rate of DCF fluorescence in macrophage cells from 8–10 wk old mice. (A) Representative data (ΔF/F_0_) from macrophages of WT (C57Bl/6J, black line), p47^phox−/−^ (red line) and Nox2^−/y^ (blue line) mice showing the temporal change in DCF fluorescence upon LPS-treatment (20 ng/ml). F_0_ is the basal fluorescence taken over the first 100 seconds prior to LPS. (B) LPS-induced stimulation (boxed areas of A) significantly increased the normalized rate of DCF fluorescence (black bar) compared to pre-stimulated values (white bar). The increased intensity of DCF fluorescence was significantly higher in WT macrophages compared to macrophages from p47^phox−/−^ and Nox2^−/y^ mice. Error bars represent s.e. from the mean. Data are analyzed from *n*_animals_ = 6 per strain, 6 replicates per animal. * p<0.05 (Tukey statistical analysis for p value).

Using the non-specific ROS indicator DCFH we found that stimulation of primary spleen macrophages with LPS increased ROS production in cells from wild-type mice, which was significantly slowed in cells isolated from Nox2 deficient (Nox2^−/y^) and completely inhibited in cells from p47^phox^ deficient (p47phox^−/−^) mice (Fig. 3A, Table 1). The small amount of ROS in the Nox2^−/y^ macrophages may be due to Nox1 activation. There was no difference between WT, Nox2^−/y^, and p47^phox−/−^ non-stimulated time controls (Fig. 3B), thus for clarity we only show the WT time control (Fig. 3A). DCFH is a single wavelength dye that shows unequal loading into cells. Therefore in order to compare rates between cells we normalized the time-course data to the maximum rate upon oxidation with H_2_O_2_. Doing so we found that LPS stimulation significantly increased ROS production only in WT macrophages and not in Nox2^−/y^ nor p47^phox−/−^ macrophages (Fig. 3B).

**Table 1:** Temporal response of DCF and p47-roGFP to LPS.

	Time (min) from application of LPS	
Cell Line	DCF	p47-roGFP
WT	8.2	3.4
Nox2^−/y^	17.2	ND
p47phox^−/−^	ND	4.6

ND: Never different.

**Figure 4:**
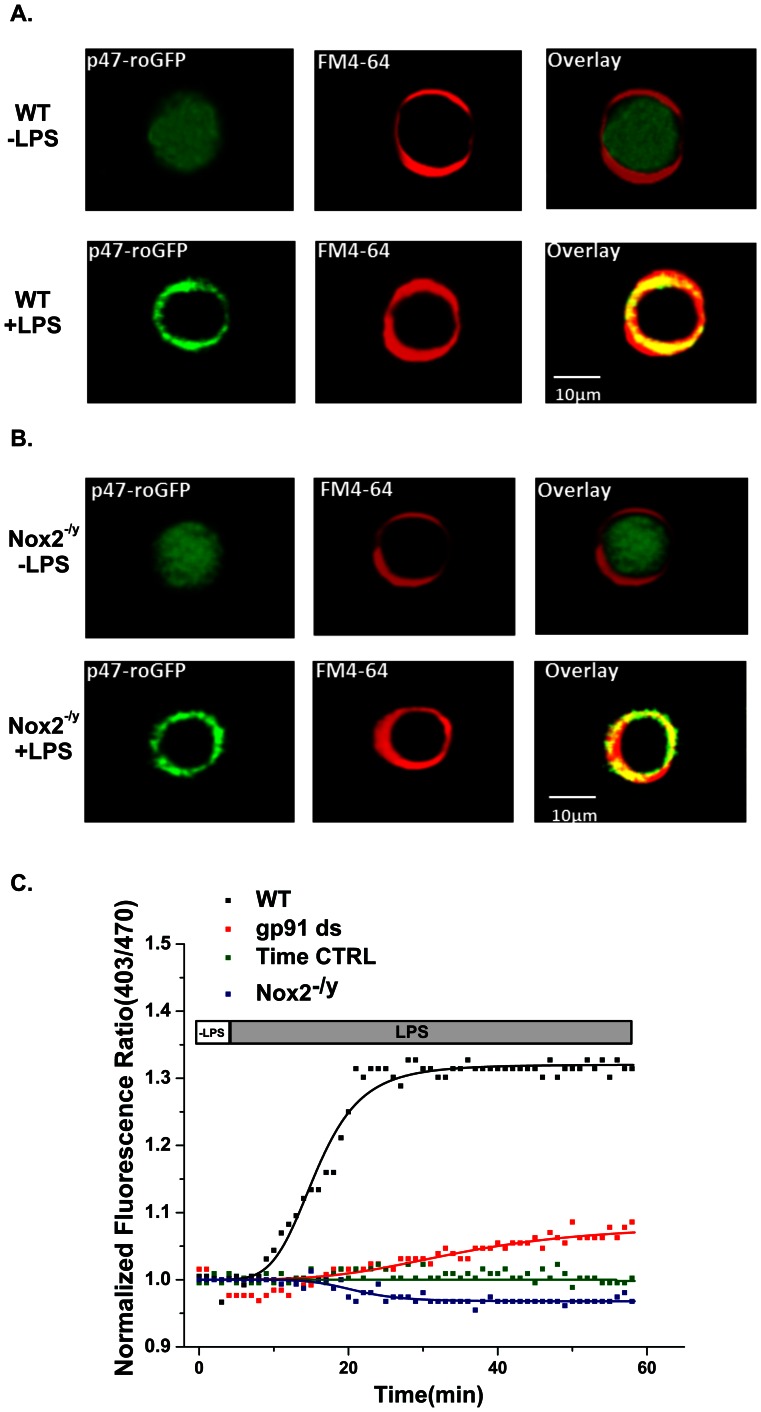
p47-roGFP biosensor shows specificity to the Nox complex in primary spleen macrophages. (A) Macrophage cells from wild-type mice (C57Bl/6j) expressing transiently transfected p47-roGFP showed homogeneous cytosolic distribution of p47-roGFP in the absence of LPS-mediated activation (−LPS). Upon LPS-mediated activation p47-roGFP localized with the membrane dye FM4-64® (+LPS). (B) In the absence of LPS (−LPS), p47-roGFP is distributed homogeneously throughout the cytosol of macrophages isolated from Nox2 deficient (Nox2^−/y^) mice. Upon LPS stimulation (+LPS), p47-roGFP translocated to the cell membrane of Nox2^−/y^ macrophages, localizing with FM4-64®. (C) LPS stimulation of primary spleen macrophages resulted in rapid oxidation of p47-roGFP in wild-type macrophages (black squares). Cells incubated with gp91ds (5 µM, 60 min.) demonstrated a significant reduction in the rate and extent of p47-roGFP oxidation (red squares). In Nox2^−/y^ macrophages (blue squares) LPS did not induce oxidation of p47-roGFP. There was no oxidation of p47-roGFP in the absence of LPS (Time CTRL, green squares). Error bars represent s.e. from the mean. Representative images (A & B) are shown from at least 9 cells. Data (C) are representative of *n*_animals_ = 6 per strain, 3 cells per animal. ** p<0.01, * p<0.05 (Tukey statistical analysis for p value).

We further characterized the specificity of p47-roGFP in macrophages harvested from wild-type and Nox2^−/y^ mice. LPS-induced stimulation (20 ng/ml) resulted in translocation of the biosensor from the cytosol to the cell membrane in both wild-type and Nox2^−/y^ primary macrophages (Fig. 4A–B). However, LPS-induced oxidation of p47-roGFP was completely prevented in the Nox2^−/y^ macrophages as well as in wild-type macrophages in the presence of gp91ds (Fig. 4C). The response of p47-roGFP to LPS-induced activation was found to be faster than that of DCF (Table 1). Taken together, our observations demonstrate the specificity of p47-roGFP toward the Nox microdomain within the cell membrane.

### p47-roGFP localizes to the endogenous Nox2 complex in skeletal muscle fibers

**Figure 5:**
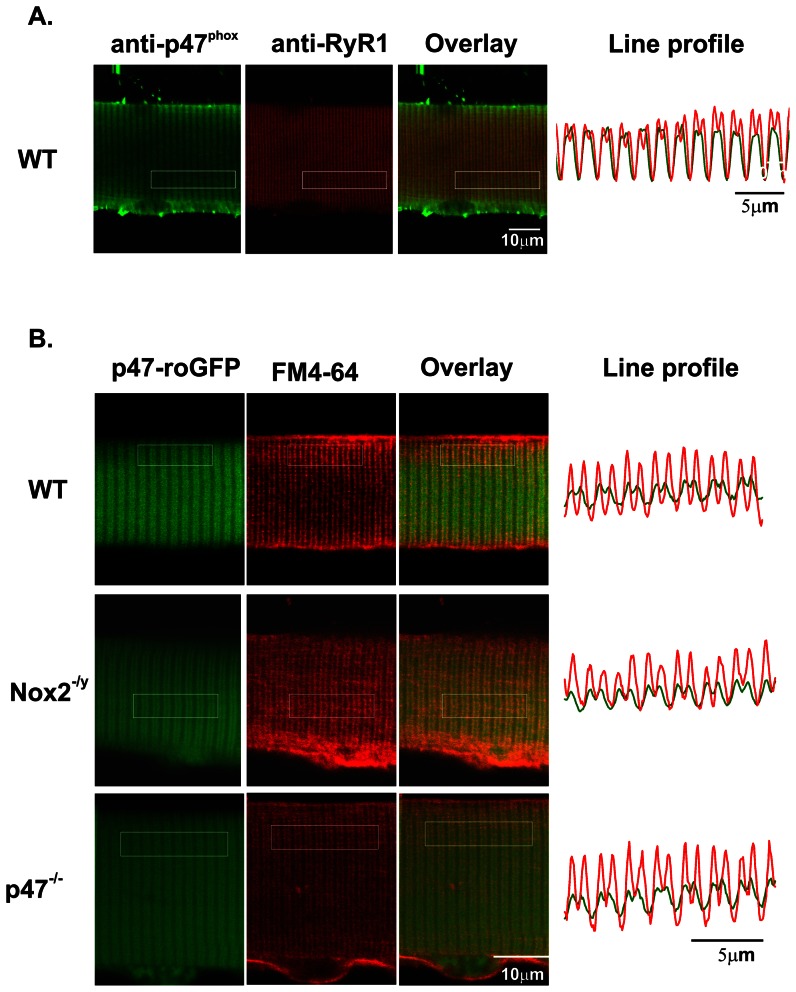
Localization of p47-roGFP in skeletal muscle. (A) Immunostaining of enzymatically dissociated single FDB myofibers for endogenous p47^phox^ and the ryanodine receptor shows that p47^phox^ co-localizes with the ryanodine receptor at the triad. (B) Fluorescent live cell image of an FDB electroporated with p47-roGFP and counter stained with the membrane and t-tubule dye FM4-64® shows that p47-roGFP is localized at the t-tubule. The line plots represent the longitudinal spatial profile of fluorescence averaged over the transverse direction within the boxed regions.

Skeletal muscle expresses only 2 isoforms of Nox, Nox2 and Nox4 [15]. The Nox2 complex has been shown to be localized to the sarcolemma and to invaginations of the sarcolemma called the transverse tubules [16] (Fig. 5A), while Nox4 is thought to be localized to the sarcoplasmic reticulum [17]. Both the voltage dependent calcium channel (CaV1.1) and the sarcoplasmic reticulum calcium release channel (ryanodine receptor, RyR1) are located at this critical site for muscle excitation-contraction coupling. Using the plasma membrane dye FM4-64® we found that electroporation of p47-roGFP into the mouse FDB resulted in localization of the biosensor at the sarcolemma and transverse tubules, the same site of the endogenous Nox2 complex. This localization occurred in muscle fibers from WT, Nox2^−/y^, and p47^phox−/−^ mice (Fig. 5B).

### Nox2 dependent ROS formation in response to physiological perturbations in skeletal muscle

**Figure 6:**
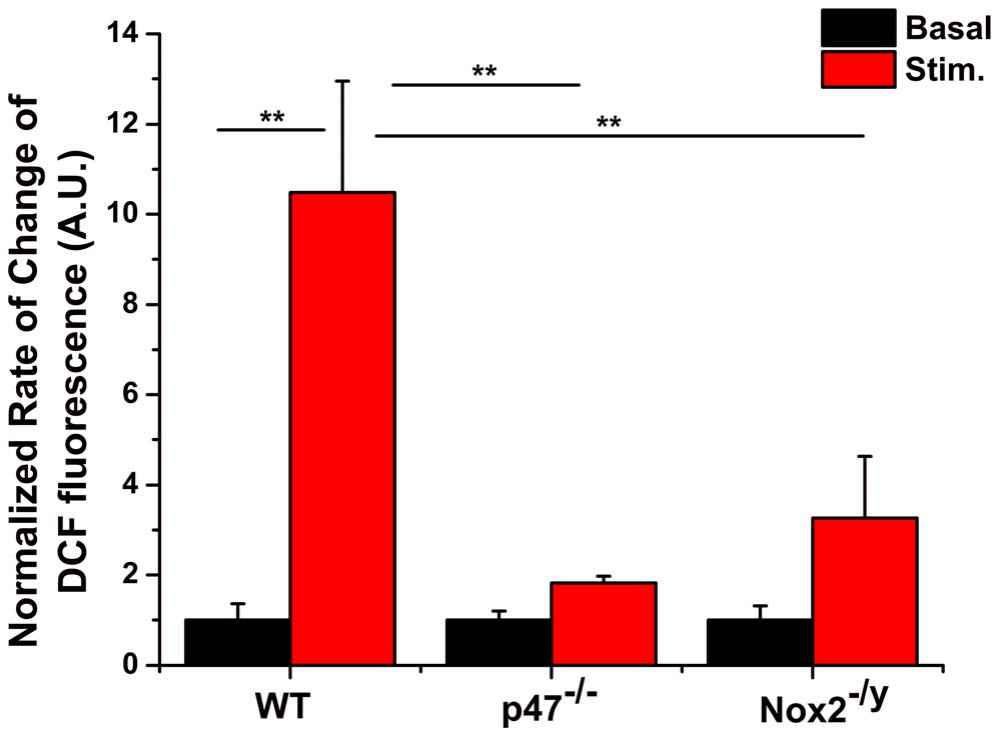
Electrical stimulation induces oxidation of DCF in myofibers from WT but not p47^−/−^ or Nox2^−/y^ mice. Tetanic electrical stimulation increased the normalized rate of DCF fluorescence (red bar) compared to pre-stimulated values (black bar). In addition, the rate of change of DCF fluorescence was significantly lower in FDB fibers from p47^phox−/−^ and Nox2^−/y^ mice compared to WT FDB fibers. There was no difference between non-stimulated and stimulated condition for either p47^−/−^ or Nox2^−/y^. Data are analyzed from *n*_animals_ = 6 per strain, 12 replicates per animal. ** p<0.01 (Tukey statistical analysis for p value).

**Figure 7:**
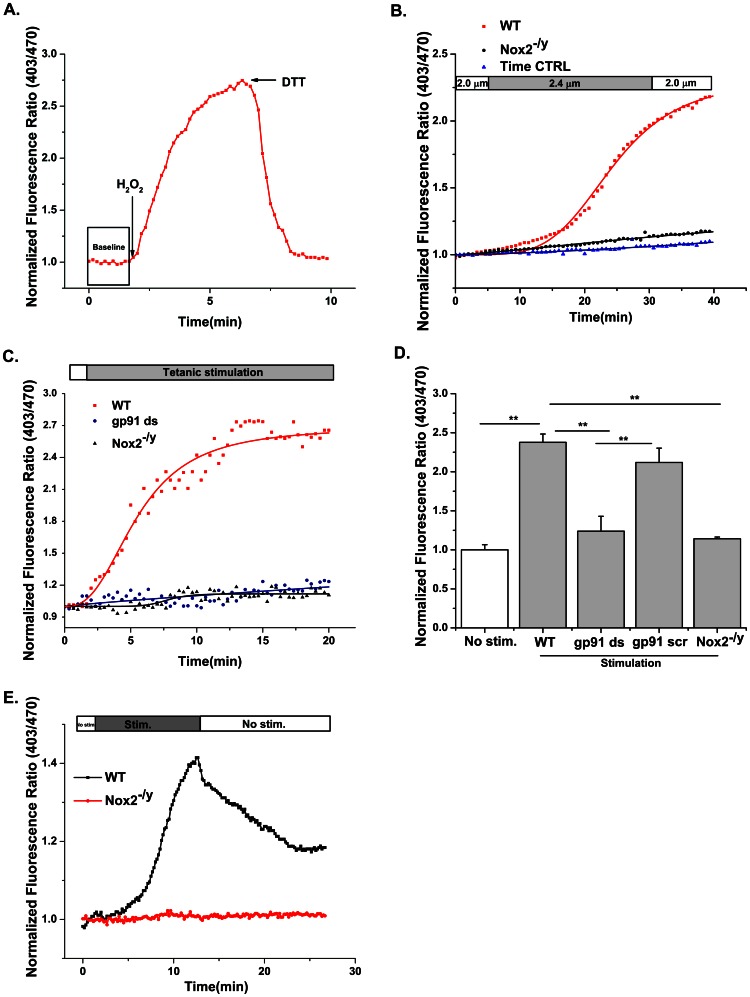
Physiological perturbation facilitates Nox2-dependant ROS formation in skeletal muscle. (A) Oxidation (H_2_O_2_, 1mM) and reduction (DTT, 10mM) of fibers expressing p47-roGFP with exogenous agents show reversibility of p47-roGFP. (B) Passive stretch to 2.4 µm (20% increase in sarcomere length) increased the oxidation state of p47-roGFP in wild-type (WT) myofibers (black squares) but not in myofibers from Nox2^−/y^ mice (black circle). (C) Repetitive contractile activity resulted in rapid oxidation of p47-roGFP sensor in WT fibers, which plateaued around 15 min. (black square). Oxidation of p47-roGFP was significantly attenuated in WT fibers treated with the Nox peptide inhibitor gp91ds (5 µm, 60 min (open circles). FDBs from Nox2^−/y^ mice also showed very minimal oxidation (black circle) of p47-roGFP. (D) Average data from experiments in (C) during the first 2 minutes (No stim.) and during the last 3 minutes of stimulation. (E) The oxidation of p47-roGFP was reversible after cessation of contraction. Data are from n_animals_ = 6 per strain, 3 cells per animal. * p <0.05, ** p <0.01 (Tukey statistical analysis for p value).

Healthy skeletal muscle produces a variety of ROS both at rest and in response to stretch and contractile activity, using highly regulated signaling pathways. We have previously shown that Nox2 is the likely source of ROS during contractile activity [7]. Using DCFH we now show decreased ROS production in myofibers from p47^−/−^ and Nox2^−/y^ mice, highlighting the crucial contribution of Nox2 in skeletal muscle ROS production during repetitive contractile activity (Fig. 6). Addition of exogenous H_2_O_2_ (1 mM) and DTT (10 mM) shows that p47-roGFP oxidation is reversible when expressed in skeletal muscle fibers (Fig. 7A). Passive stretch of single FDB myofibers to ∼120% (2.4 µM) of resting length resulted in oxidation of p47-roGFP (Fig. 7B). In addition, repetitive contractile activity resulted in rapid oxidation of p47-roGFP, which plateaued before the end of the contractions (Fig. 7C). p47-roGFP measured ROS produced specifically from Nox, as inhibition of Nox with gp91ds or removal of Nox2 (Nox2^−/y^) completely prevented oxidation of the biosensor in response to passive stretch (Fig. 7B) and contractile activity (Fig. 7C–D, Table 1). Upon halting the electrical stimulation p47-roGFP redox state rapidly returned back toward the pre-stimulated reduced state (Fig. 7E). Experiments are currently underway to determine why p47-roGFP redox balance did not return completely to the baseline value.

**Figure 8:**
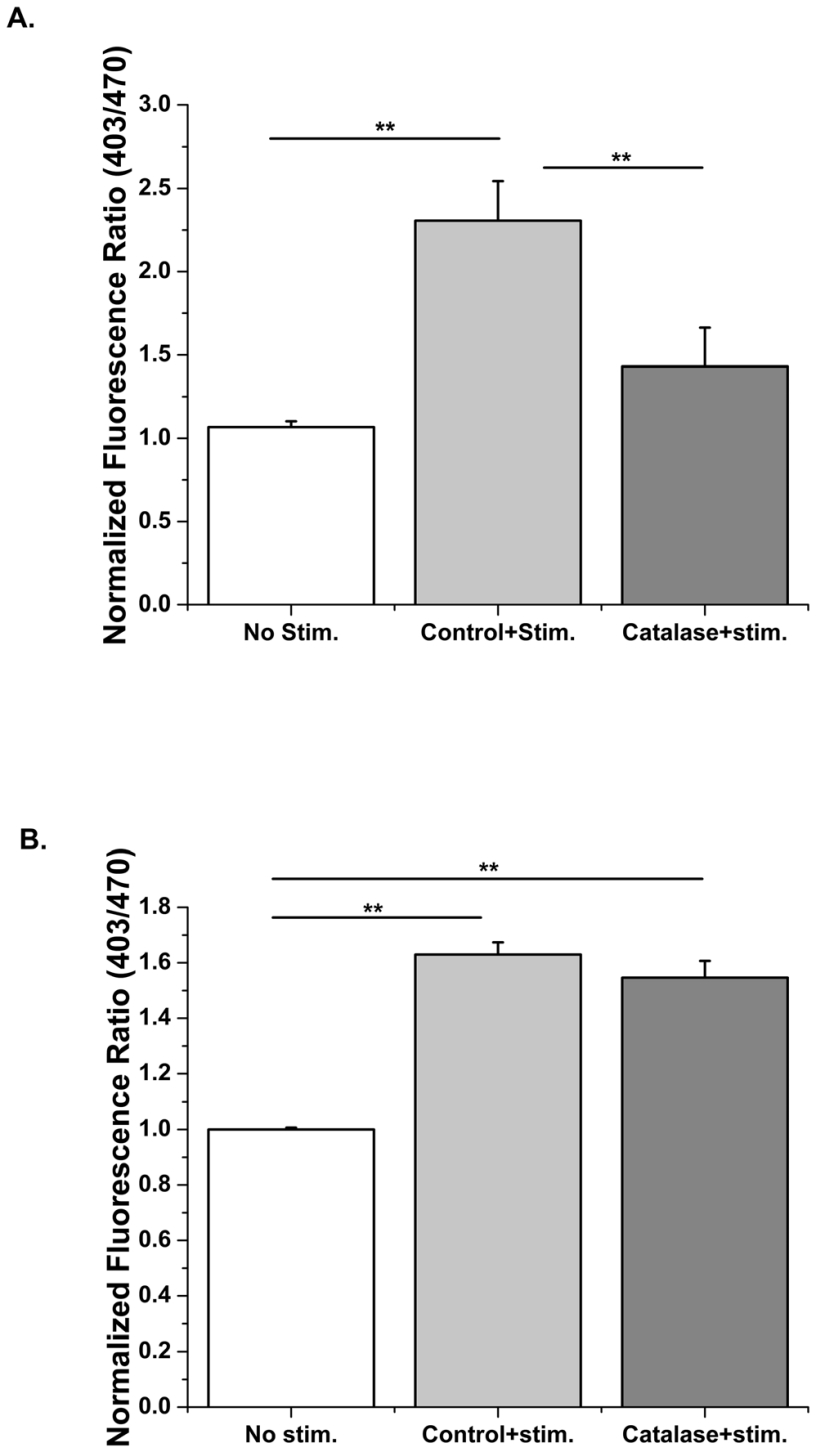
p47-roGFP measures Nox dependent ROS produced in the extracellular space. Catalase (4 µM) in the extracellular space prevented the oxidation of p47-roGFP (A) but did not alter the oxidation of Grx1-roGFP2 (B) during electrical stimulation of FDB myofibers.

In macrophages Nox produces superoxide into the extracellular space, which rapidly dismutes into H_2_O_2_ and O_2_ that diffuse across the plasma membrane. Therefore, we tested whether activation of Nox2 in skeletal muscle analogously produces ROS in the extracellular space. Addition of catalase (4 µM) into the extracellular space prevented the electrical stimulation induced oxidation of p47-roGFP (Fig. 8A), indicating that the oxidation of p47-roGFP is completely dependent upon ROS production from Nox. While extracellular catalase prevented oxidation of p47-roGFP, it did not prevent oxidation of roGFP2 linked to glutaredoxin 1 (Grx1-roGFP2, Fig. 8B). These data show that p47-roGFP is not responding to local changes in the GSH/GSSG, as does Grx1-roGFP, but preferentially detects ROS generated from Nox.

### p47-roGFP rescues Nox activity in p47 deficient cells

**Figure 9:**
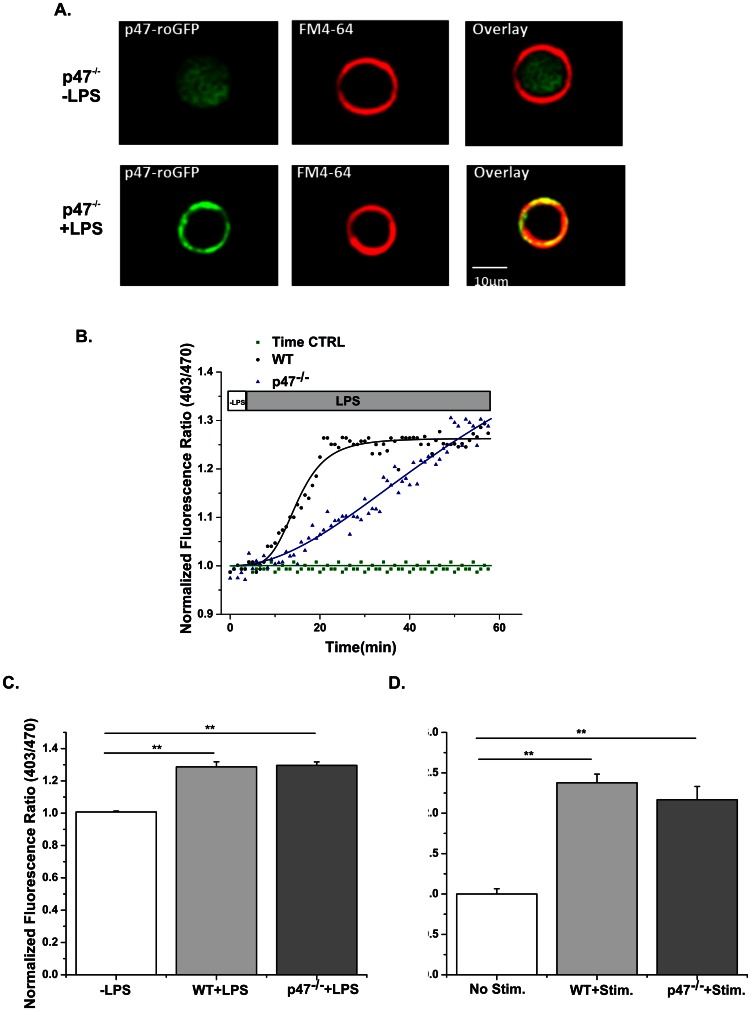
Nox-mediated ROS formation is rescued by p47-roGFP in p47^phox−/−^ cells. (A) Macrophage cells from p47^phox−/−^ mice transiently transfected with p47^phox^- showed homogeneous cytosolic distribution of p47-roGFP in the absence of LPS-mediated activation (left). Cells incubated with LPS (20 ng/ml, 45 min, right) showed p47-roGFP localization with the membrane dye FM4-64® around the periphery of the cell. (B) LPS stimulation of primary macrophages resulted in rapid oxidation of p47-roGFP in wild-type macrophages (WT, black squares). p47^phox−/−^ cells showed a very similar level of p47-roGFP oxidation, but with a slower rate (blue squares). In the absence of LPS-stimulation, wild-type cells showed almost no change in oxidation of p47-roGFP sensor (green square). (C) Average data from (B) before addition of LPS (−LPS) and during the last 3 minutes of LPS stimulation (+LPS). (D) Tetanic electrical stimulation of single FDBs from wild-type and p47^phox−/−^ mice expressing p47-roGFP resulted in similar levels of p47-roGFP oxidation compared to WT. Representative images (*A*) are shown from at least 9 cells. Data (B–C) are representative of *n*_animals_ = 8 per strain, 3 cells per animal and for (*E*) *n*_animals_ = 4 per strain, 3 cells per animal. ** p<0.01 (Tukey statistical analysis for p value).

To further test whether our Nox biosensor, p47-roGFP, is an active component of the Nox ROS generating complex, we assessed its ability to rescue Nox activity in a mouse lacking p47^phox^ (p47^phox−/−^). Transfection of primary macrophages isolated from p47^phox−/−^ mice with p47-roGFP showed translocation of the biosensor from the cytosol to the membrane (Fig. 9A) and increased ROS production (Fig. 9B–C) upon activation with LPS. In single p47^phox−/−^ FDB skeletal muscle fibers, p47-roGFP was sufficient to rescue Nox2 dependent ROS production in response to contractile activity (Fig. 9D). However, the sensor responded with a slower oxidation rate in p47^phox−/−^ animals compared to the WT animals (Table 1), which is likely due to decreased levels of total p47^phox^ (endogenous plus p47-roGFP) in p47^phox−/−^ animals. Taken together, the p47-roGFP Nox biosensor behaves as endogenous p47^phox^ and detects ROS specifically from Nox.

## Discussion

Defining the role of ROS in cell biology has been limited by our inability to identify the sub-cellular sites of ROS production, the signaling mechanisms that regulate their production, and whether these sites differ under physiological versus pathophysiological conditions. In order to develop inhibitors that target a specific source of ROS, redox sensors should be non-disruptive, dynamic, sensitive, and compartmentally specific. Over the last few years fluorescent protein probes have been utilized to measure changes in the redox environment within the cell. Hyper (cpYFP-OxyR) was developed as a specific probe for H_2_O_2_; however it is sensitive to changes in pH [18], [19] and therefore caution has to be taken to ensure any observed change is due to redox dependence and not pH. The redox sensitive GFP (roGFP2) is a ratiometric probe that has been fused to the peroxidase Orp1 (Orp1-roGFP2) [20] for the measurement of H_2_O_2_ as well as glutaredoxin 1 (Grx1-roGFP2) [10] for measuring glutathione redox potential [10]. Unlike Hyper, the roGFP2 ratio is insensitive to changes in pH [10], [21]. More recently, two FRET-based ROS sensors (OxyFRET and PerFRET) have been used to measure H_2_O_2_ within subcellular compartments [22]. While Hyper and roGFP2 show a 3–9 fold change in their ratio upon pharmacological oxidation with H_2_O_2_, both OxyFRET and PerFRET exhibit minimal fluorescence changes of 1.0 and 1.2 fold, respectively.

In this study we have fused roGFP2 to p47^phox^ to generate a redox biosensor that allows for noninvasive quantitative imaging of NADPH oxidase activity. Thus far seven Nox isoforms (Nox1, -2, -3, -4, 5, Duox1, -2) have been shown to be expressed in mammalian cells. The Nox2 complex has recently gained attention as a major contributor of ROS production in a multitude of cell types. The activation of the plasma membrane subunits gp91^phox^ (Nox2) and p22^phox^ occurs through a complex series of protein/protein interactions. p47^phox^ is designated as the “organizer subunit” and thus facilitates the translocation of other cytosolic factors to the plasma membrane. p47^phox^ binds both Nox2 and p22^phox^ and is essential for Nox2 activation. Here, we show that the expression of p47-roGFP allowed for real-time assessment of Nox activity in living cells in response to exogenously applied agents as well as physiological conditions associated with immune cell activation and increased skeletal muscle activity.

A critical feature of an exogenously expressed reporter protein is that it behaves like the endogenous protein. We found that upon LPS induced activation of immune cells p47-roGFP translocated from the cytosol to the plasma membrane, where it was oxidized. This oxidation was reversible upon reduction with DTT. We found that the Nox peptide inhibitor (gp91ds) inhibited LPS induced oxidation of p47-roGFP in both RAW264.7 and primary macrophages. In addition, despite translocation of p47-roGFP to the plasma membrane, where it can bind p22^phox^, in Nox2^−/y^ macrophages upon LPS stimulation, the oxidation state of p47-roGFP did not differ from that prior to LPS. When expressed in macrophages from p47^phox−/−^ mice, p47-roGFP was able to promote Nox activation and ROS production, albeit at a slower rate than in macrophages from WT mice. It is unclear as to why we observed a slower rate of p47-roGFP oxidation in p47^phox−/−^ macrophages, but it could be due to enhanced activation of Nox in cells overexpressing p47^phox^ [23]. Alternatively, altered interactions between p47-roGFP and the accessory proteins it organizes and recruits to the membrane (i.e. p67^phox^, Rac) may explain the difference. Experiments are currently underway to understand this phenomenon. Taken together, these data strongly suggest that p47-roGFP is a dynamic biosensor that specifically detects NADPH oxidase activity.

Repetitive electrical stimulation increases ROS production in skeletal muscle [7], [24]–[27]. Using a redox sensitive GFP targeted to the mitochondria along with pharmacological inhibitors we have previously shown that the source of ROS during electrical stimulation is likely Nox [7]. Using our novel Nox targeted redox biosensor we now show that Nox dependent ROS production contributes significantly to the increased oxidation state of contracting skeletal muscle. Oxidation of p47-roGFP was inhibited by gp91ds and absent in Nox2^−/y^ muscle fibers. Stopping the electrical stimulation resulted in an initial rapid reduction of p47-roGFP fluorescence, showing that p47-roGFP responds quickly and robustly to activation and inactivation of Nox. The probe did not return to baseline within the time frame of the experiment, perhaps reflecting a slower return of the whole cell redox balance, which the probe is likely able to track (see discussion below). Furthermore, p47-roGFP was able to replace p47^phox^ in myofibers from p47^phox−/−^ mice. Khairallah et al [28] have recently shown ROS production in response to passive stretch only under pathological conditions. Here we show, for the first time, activation of Nox in response to passive stretch under non-pathological conditions. The likely difference between our results and those by Khairallah and colleagues lies in the method of ROS detection. In the previous study the authors used DCFH, which does not provide subcellular specificity and has low signal to noise ratio. In this study we were able to direct our redox probe directly to the source of the ROS, dramatically increasing the sensitivity and specificity of the biosensor.

Linking roGFP2 to p47^phox^ has allowed for subcellular targeting of a redox sensitive fluorescent protein reporter directly to NADPH oxidase. Macrophages express both Nox1 and Nox2. While Nox1 is primarily activated by NOXO1 (homologue of p47^phox^) [1], [29], p47^phox^ can activate Nox1 in heterologous expression systems [30]. Nox3 can also be activated by p47^phox^, thus p47-roGFP may be used to effectively monitor Nox1, -2, or -3 activity. We found that p47-roGFP was not significantly oxidized upon LPS activation of macrophages nor during either passive stretch or electrical stimulation of skeletal muscle from Nox2^−/y^ mice. In skeletal muscle only Nox2 and Nox4 are expressed [15]. Taken together, these data suggest that p47-roGFP shows specificity toward Nox2 activity. The mechanism by which p47-roGFP confers specificity toward Nox2 activity is currently under investigation.

In a recent report by Enyedi et al the authors suggest that by targeting OxyFRET and PerFRET to the plasma membrane and using diphenyleneiodonium (DPI, a flavoenzyme inhibitor) they are able to detect Nox dependent ROS generation [22]. They suggest that PMA stimulation of granulocyte-like cells activates Nox dependent ROS production, which penetrates to the mitochondria. Both the plasma membrane and mitochondrial targeted probes are remarkably oxidized at the same rate and are both inhibited by DPI at concentrations that have been shown to inhibit not only Nox but also NADH-ubiquinone oxidoreductase (mitochondrial complex I) [31]. In our studies we target roGFP2 to the Nox enzyme complex through p47^phox^. We show activation, translocation, and oxidation of p47-roGFP upon physiological stimulation. p47-roGFP oxidation is inhibited by the Nox specific peptide inhibitor and is absent in cells from Nox2^−/y^ mice. Scavenging of extracellular H_2_O_2_ with catalase prevented the oxidation of p47-roGFP but not roGFP2 linked to glutaredoxin (Grx1-roGFP2). These findings suggest that p47-roGFP is not solely detecting changes in GSH/GSSG, as does Grx1-roGFP2, but is oxidized by Nox dependent ROS generated in the extracellular space that diffuses back across the membrane. The most likely species diffusing back across the membrane and oxidizing p47-roGFP is H_2_O_2_, but at this time we cannot rule out that other oxidants (i.e. hydroxyl radical or lipid peroxides) are also able to oxidize the probe. It is likely that p47-roGFP can still respond to cellular redox changes, which may explain the incomplete return to baseline after removal of the electrical stimulation in skeletal muscle. However, by targeting it to the subcellular microdomain where the Nox enzyme complex resides we have been able to improve the performance of the biosensor and provide greater specificity to Nox dependent ROS production.

### Conclusion

We have generated a redox biosensor that allows for real-time measurements of NADPH oxidase activity in living cells. In principle, such specificity in measurements can be applied to any physiological and pathological condition in a variety of living systems; highlighting the applicability of our biosensor to drug screening and toxicology. As evidence suggesting that the Nox complexes play important roles in the signal transduction of various cellular stress responses has accumulated, and the recent efforts in identifying specific Nox inhibitors have escalated, our novel Nox biosensor greatly advances the field of redox biology, potentially contributing to the discovery of specific therapeutic drug targets.
